# Radically Embodied Compassion: The Potential Role of Traditional Martial Arts in Compassion Cultivation

**DOI:** 10.3389/fpsyg.2020.555156

**Published:** 2020-09-25

**Authors:** Neil Clapton, Syd Hiskey

**Affiliations:** ^1^Avon and Wiltshire Mental Health Partnership NHS Trust, Swindon, United Kingdom; ^2^Private Practice, The Oaks Hospital, Colchester, United Kingdom

**Keywords:** martial arts, compassion, radically embodied, polyvagal theory, conflict resolution

## Introduction

The last few years have seen a growing interest and research into the science of compassion, including exploring how we can best cultivate this important and powerful affiliative/prosocial motivation and its behavioral manifestations in humans (Gilbert, 2019). This has resulted in a proliferation of various compassion cultivation programmes and interventions (Kirby, 2017; Kirby et al., 2017), both for clinical and non-clinical populations. Many of these programmes contain both similarities and differences in regards to the specific mind training practices they draw on to stimulate the proposed neurophysiological processes associated with compassionate motivation and behaviors (Gilbert, 2019; Kim et al., 2020).

Whilst Western science has begun to consider ways of more fully embodying compassion (Loizzo, 2018; Khoury, 2019), it has thus far failed to consider the potentially powerful role of ancient cultural practices such as the traditional martial arts in cultivating compassion. This is despite the fact that the purpose and philosophy of many traditional martial arts is to develop certain virtuous character strengths (Hackney, 2011) in order to become a fearlessly courageously compassionate human being (Kamen, 2017). This opinion paper shall briefly outline the rationale for and extol the potential benefits of the traditional martial arts in entraining compassion at a radically embodied level (Hiskey and Clapton, 2019a), including related research that already supports this theoretical proposition.

## Martial Arts as Radically Embodied Compassion

### The Nature of Compassion and Its Neurophysiological Correlates and Indicators

Whilst debates remain about the nature of and how to define compassion (Gilbert, 2017; Khoury, 2019), there is growing consensus that compassion is most usefully defined as “a sensitivity to suffering in self and others, with a commitment to try to alleviate and prevent it” (Gilbert and Choden, 2013, p. 94). A commonality among evolutionary, motivational and Buddhist psychology is to see compassion as a *motive*, likely rooted in evolved caring and affiliative motivational systems (Gilbert, 2015, 2017, 2019). From this perspective, at the core of compassion is *courage* and *wisdom*, in terms of (1) courageous willingness and emotional strength to turn toward, empathically engage with and tolerate distress; and (2) dedication to learn and develop skills/skillful means to take wise action in alleviating and preventing/halting suffering.

At a neurophysiological level, Polyvagal Theory (Porges, 2007, 2011) proposes that compassion is associated with and dependent on a vagally-mediated physiological state and activation of the parasympathetic nervous system (Porges, 2017; Stellar and Keltner, 2017) that is functionally distinct from empathy and affords feelings of personal *safety*, as a result of simultaneously encompassing the feeling of one's own bodily responses while acknowledging those of the other (Porges, 2017).

Key to compassion then is that it allows one to stay engaged with suffering without becoming overwhelmed or overly distressed by it (Wilson and Takuan, 2012), reducing fear whilst affording and sustaining compassionate responding in difficult and challenging situations (Ashar et al., 2019).

### Radically Embodied Compassion as Understood, Cultivated and Entrained by the Traditional Martial Arts

Compassion is a central tenet, ethical intention and motivational force integral to the “Way” of many traditional martial arts, including aikido (Westbrook and Ratti, 1970), taekwondo (Cook, 2006) and karate (Kamen, 2017). Traditional martial arts philosophy and systems are commensurate with the aforementioned conceptualization of compassion, as a motive to empathically confront and take wise skillful action to reduce and prevent suffering of self and others (Ueshiba, 2007). At their core, traditional martial arts are mind-body self-cultivation systems (Sovereign and Walker, 2020) that help develop compassionate and virtuous character strengths such as courage, benevolence, wisdom, temperance and justice (Hackney, 2011, 2013). Seen from this perspective, martial arts can become a vehicle and pathway to reducing suffering, promoting well-being and flourishing at a personal and social level (Hackney, 2013; Sovereign and Walker, 2020).

The neuroscience of embodiment proposes and elucidates the centrality of the body in influencing and shaping motivational, emotional and cognitive processes (Price et al., 2012). Experimental evidence has demonstrated how intentionally-manipulated bodily states, such as dynamic/static body posture and quality/shape of movement, influence physiological responses associated with approach and avoidance motivations, emotional processing and perception (Price et al., 2012; Osypiuk et al., 2018). Such theory and research helps to provide an explanatory framework as to how and why traditional martial arts may be a form of *Radically Embodied Compassion*. Our proposition is that traditional martial arts training involves harnessing and training the *whole* self via a multitude of integrated mind-body practices that stimulate/give rise to compassionate motivation and associated mental states, emotions and behaviors, in the service of halting or reducing suffering.

Existing research already supports the notion that traditional martial arts afford, entrain and stimulate (neurophysiological) processes that are associated with and underpin compassionate motivation and compassionate responding (Kim et al., 2020). Such training aims to develop ease of access to a psychophysiological state characterized by courageous, swift, and precise responsiveness in the face of threats (Faggianelli and Lukoff, 2006). Thus, one can respond skillfully and flexibly from a position of inner centeredness, relative calm and stability (Martin, 2004; Wilson and Takuan, 2012), a state of mind that is reflective of and affords wise courageous compassionate engagement and action (Ueshiba, 2007). For example, rather than flinching or turning away in the face of physical confrontation, one might enact entrained procedures to block and counter it with one's own skillful actions. Recent fMRI research into the effects of mind-body training through *Budo* in Kendo practitioners on the motivation network supports this notion, in that “resting vs. attentionally-driven” switching/change of motivation can be trained and becomes more efficient (Fujiwara et al., 2019), which is indicative of an unmoving/undisturbed/unfettered mind (Wilson and Takuan, 2012) which better affords an appropriate physical response. Research also suggests that martial artists are more endogenously prepared to engage with and respond to uncertainty (Johnstone and Marí-Beffa, 2018) and are more distress tolerant when faced with and despite repeated exposure to threatening situations (Staller et al., 2017), as well-having greater sustained attentional abilities and impulse control (Sánchez-López et al., 2013; Sánchez-Lopez et al., 2016). All of this combines to lead to skillful action that is reasoned, assertive and conflict-ending rather than mindlessly aggressive and designed to excessively hurt another.

The power of martial arts as a mind-body practice and discipline potentially lies in their ability to foster optimal sympathovagal balance that affords flexible and optimal compassionate responding. Similar other mind-body practices such as Tai Chi, Qigong and yoga that pair mindful movement with breathing/breath control exercises have been shown to stimulate the parasympathetic nervous system (Ramos et al., 2017; Sullivan et al., 2018; Walther et al., 2018) and many martial forms/kata have sections that are deliberately practiced in a slow, controlled and meditative way (Rawcliffe, 2003). Paced rhythmic breathing synchronized with rhythmic muscle contraction activates and produces a more resilient parasympathetic response in the face of stress than either alone (Chin and Kales, 2019), both of which are integral to martial arts practice and are often seen in prearranged practice drills (i.e., Kihon), all conducted with respect for oneself and one's partner.

Research into behavioral synchrony is directly relevant to the processes which are stimulated by martial arts training. Group activities where people engage in synchronized movements, expressive gestures and collective rituals such as those found in martial arts training have been shown to enhance compassion for others via processes of shared flow and perceived emotional synchrony (Pizarro et al., 2020). Such collective behavioral synchrony has been proposed to help cultivate and enhance compassion because it likely co-stimulates affiliative and co-operative social mentalities (Friedman, 2016; Gilbert, 2017) that facilitates reciprocal empathic and altruistic responding (Valdesolo and DeSteno, 2011; Pizarro et al., 2020) even when enacting confrontation related (i.e., fight) behavior.

All martial arts involve some form of carefully managed and safe combat (i.e., sparring or Kumite), whereby two or more practitioners are engaged in a dynamic process of antagonistic engagement, skillful responding and co-regulation (Kimmel and Rogler, 2019) that ends in reaffiliation and mutual respect at the end of the encounter/confrontation (e.g., bowing or touching gloves). Importantly, these are dyadic *prosocial* encounters (Rassovsky et al., 2019) that require moving from asynchronous to synchronous interactions and states. Such encounters require martial artists to rapidly and flexibly shift between socio-motivational states, otherwise referred to as Locomotion Motivation (Webb et al., 2017), an ability which is crucial in facilitating conflict resolution, post-conflict reconciliation, learning and growth (Webb et al., 2017). Martial arts might be implicitly and explicitly entraining value-driven abilities to stay affiliatively engaged in conflict situations of high relational threat and end such conflicts by reaffiliating, with minimum harm done. Thus, martial artists develop the courage and confidence to face and resolve conflicts non-violently and compassionately, whereby potential suffering is halted and prevented and relational harmony is restored. This is compassionate conflict resolution and transformation (Martin, 2004; Friedman, 2016; Lukoff and Strozzi-Heckler, 2017; Hiskey and Clapton, 2019a).

Sparring also often involves some form of physical touch and contact either via blocking and/or grappling. Such martial arts training has been shown to increase endogenous oxytocin production (Rassovsky et al., 2019), a neuropeptide that plays a central role in regulating mammalian social behaviors by down-regulating threat-based processing and defensive behaviors and up-regulating affiliative and prosocial behaviors associated with compassion. Taken together, traditional martial arts training and practices maybe a particularly powerful way of neurally exercising vagal circuits and pathways of what has been termed the Social Engagement System (Porges, 2017).

Crucially, such compassion and compassionate abilities cultivated and entrained by the martial arts are likely transferable to non-martial situations, through a process which has been termed *somatic metamorphism* (Foster, 2015). This has been described as a mode of deploying the body to make sense of and wisely respond to non-martial challenges such as day-to-day relational conflicts and challenges, supported by first-person accounts of Aikido practitioners' somatic metamorphism in everyday life (Foster, 2015). In this way, martial values of care for ourselves and others generalize from the dojo/training hall to one's broader relational context(s).

### Clinical and Non-clinical Applications of Martial Arts-Based Compassion Cultivation

The potential utility of martial arts as a vehicle for cultivating compassion is broad and multifarious. In terms of clinical applications, there is emerging theory that entraining radically embodied compassion in therapists/clinicians via traditional martial arts may afford them greater distress tolerance and compassionate responsiveness in difficult therapeutic encounters such as alliance ruptures (Faggianelli and Lukoff, 2006; Hiskey and Clapton, 2019a,b), where repair of and reaffiliation after such ruptures are central to good therapeutic outcomes (Safran et al., 2002; Eubanks-Carter et al., 2015). Martial arts-augmented personal practice thus has the potential to help develop specific compassionate competencies that not only benefit others but also the self (Twemlow, 2001a,b; Hiskey and Clapton, 2019a,b) by enhancing the flow of compassion (Gilbert et al., 2017), as well as acting as an important form of compassionate self-care for mental health professionals (Gladstone, 2018).

Traditional martial arts training and practices also hold promise as a compassion-focused psychotherapeutic intervention in their own right and/or as an adjunct to other psychotherapies. Early evidence suggests that martial arts training is a potentially powerfully transformative intervention for individuals who have experienced interpersonal trauma (Vargas, 2019), possibly because of martial arts' highly embodied and somatic-based nature that is akin to other somatically-informed psychotherapies that target the amelioration and healing of trauma more directly via the body (Ogden and Fisher, 2015; Van der Kolk, 2015; Loizzo, 2018). Martial arts-informed psychotherapy may also be a more compassionate way to reach and help alleviate the suffering of individuals whom find it extremely difficult to access and benefit from verbal therapies alone, such as people whose traumatic histories and upbringings lead to acting out destructive aggression and a relative inability to mentalize about others or the self (Twemlow et al., 2008).

In terms of cultivating and engendering compassion at a social level, traditional martial arts has much to offer. It has the potential to decrease and prevent the suffering engendered by inevitable interpersonal conflicts that occur in day-to-day life, and promote more prosocial behaviors associated with compassionate motives by embodying and enacting non-violent conflict resolution and facilitated post-conflict reaffiliation (Martin, 2004; Foster, 2015; Friedman, 2016; Lukoff and Strozzi-Heckler, 2017; Hiskey and Clapton, 2019a). A growing body of research and evidence supports the notion that longer-term training in traditional martial arts reduces aggressiveness and increases prosocial behaviors (Blomqvist Mickelsson, 2019). Some of these other compassionate behaviors include the development and expression of prosocial assertiveness and helpful bystanding (Twemlow et al., 2008) as well as respect for others, perseverance, self-confidence, and healthy habits (Chinkov and Holt, 2015).

## Conclusion

This paper has outlined how the traditional martial arts may be powerful ways of cultivating radically embodied compassion. Western science has thus far largely ignored and neglected the ancient wisdom of the traditional martial arts as vehicles for compassion cultivation and training. Our hope is that this paper will stimulate further research into the underlying mechanisms of martial arts practices and their relationship to compassion, as well as the further development of martial arts-based psychotherapies and personal practices that help cultivate compassion for all.

## Author Contributions

NC and SH contributed to this topic and writing equally.

## Conflict of Interest

The authors declare that the research was conducted in the absence of any commercial or financial relationships that could be construed as a potential conflict of interest.
